# The β-catenin/YAP signaling axis is a key regulator of melanoma-associated fibroblasts

**DOI:** 10.1038/s41392-019-0100-7

**Published:** 2019-12-24

**Authors:** Tianyi Liu, Linli Zhou, Kun Yang, Kentaro Iwasawa, Ana Luisa Kadekaro, Takanori Takebe, Thomas Andl, Yuhang Zhang

**Affiliations:** 1 Division of Pharmaceutical Sciences, College of Pharmacy, University of Cincinnati, Cincinnati, OH 45267 USA; 2 Division of Gastroenterology, Hepatology & Nutrition, Cincinnati Children’s Hospital Medical Center, 3333 Burnet Avenue, Cincinnati, OH 45229-3039 USA; 3 Division of Developmental Biology, Cincinnati Children’s Hospital Medical Center, 3333 Burnet Avenue, Cincinnati, OH 45229-3039 USA; 4 Department of Dermatology, College of Medicine, University of Cincinnati, Cincinnati, OH 45267 USA; 5 Center for Stem Cell and Organoid Medicine (CuSTOM), Cincinnati Children’s Hospital Medical Center, 3333 Burnet Avenue, Cincinnati, OH 45229-3039 USA; 6 Department of Pediatrics, University of Cincinnati College of Medicine, 3333 Burnet Avenue, Cincinnati, OH 45229-3039 USA; 7 Institute of Research, Tokyo Medical and Dental University 1-5-45 Yushima, Bunkyo-ku, Tokyo 113-8510 Japan; 8 Burnett School of Biological Sciences, University of Central Florida, Orlando, FL 32816 USA

**Keywords:** Cancer microenvironment, Cell biology

## Abstract

β-catenin is a multifunctional protein that plays crucial roles in embryonic development, physiological homeostasis, and a wide variety of human cancers. Previously, we showed that in vivo targeted ablation of β-catenin in melanoma-associated fibroblasts after melanoma formation significantly suppressed tumor growth. However, when the expression of β-catenin was ablated in melanoma-associated fibroblasts before tumor initiation, melanoma development was surprisingly accelerated. How stromal β-catenin deficiency leads to opposite biological effects in melanoma progression is not completely understood. Here, we report that β-catenin is indispensable for the activation of primary human stromal fibroblasts and the mediation of fibroblast-melanoma cell interactions. Using coimmunoprecipitation and proximity ligation assays, we identified Yes-associated protein (YAP) as an important β-catenin-interacting partner in stromal fibroblasts. YAP is highly expressed in the nuclei of cancer-associated fibroblasts (CAFs) in both human and murine melanomas. Mechanistic investigation revealed that YAP nuclear translocation is significantly modulated by Wnt/β-catenin activity in fibroblasts. Blocking Wnt/β-catenin signaling in stromal fibroblasts inhibited YAP nuclear translocation. In the absence of YAP, the ability of stromal fibroblasts to remodel the extracellular matrix (ECM) was inhibited, which is consistent with the phenotype observed in cells with β-catenin deficiency. Further studies showed that the expression of ECM proteins and enzymes required for remodeling the ECM was suppressed in stromal fibroblasts after YAP ablation. Collectively, our data provide a new paradigm in which the β-catenin-YAP signaling axis regulates the activation and tumor-promoting function of stromal fibroblasts.

## Introduction

The development and progression of cancer are remarkably influenced by the tumor microenvironment (TME), which is composed of noncancer stromal cells, including endothelial cells, immune cells and fibroblasts, and noncellular components, such as growth factors, chemokines, cytokines, metabolites, and the extracellular matrix (ECM) proteins.^1^ One major source of cancer-associated fibroblasts (CAFs) in the tumor stroma is resident stromal fibroblasts.^2^ While fibroblasts surrounding hair follicles tend to be active due to hair cycling, the majority of dermal fibroblasts appear static and dormant under normal physiological conditions.^3^ It is known that stromal fibroblasts can be activated by cancer cells to initiate phenotypic, molecular, and biochemical transitions and to transdifferentiate into CAFs,^4^ which then possess unique properties for promoting tumor cell growth and metastasis.

As a major component of the TME, the ECM plays a critical role in cancer progression and therapeutic response.^5^ The ECM possesses a dynamic architecture composed of a complex mixture of macromolecules and is constantly remodeled via protein deposition, degradation, modification and crosslinking by tumor and stromal cells.^6^ CAFs generally contribute to two major ECM remodeling activities.^7^ First, fibroblasts synthesize and deposit various ECM proteins, such as type I, III and IV collagens, elastin, fibronectin, tenascin C (TNC), and proteoglycans (PGs), which are all essential components in maintaining the interconnected ECM structure. Second, fibroblasts contribute to ECM degradation and remodeling by producing different types of enzymes, such as matrix metalloproteinases (MMPs), lysyl oxidases, or lysyl hydroxylases. As such, CAFs play critical roles in depositing, degrading, and modifying matrix components to maintain a stiff tumor ECM. Nevertheless, the mechanisms by which normal stromal fibroblasts interact with cancer cells, convert into CAFs and remodel the ECM need to be further elucidated.

β-catenin is a multifunctional protein that plays crucial roles in both cadherin-based cell-cell adhesion and Wnt signaling-mediated gene expression.^8^ It has been clearly demonstrated that the canonical WNT/β-catenin signaling pathway is associated with fibroblast activation, fibrosis, and tissue repair.^9^ Previously, we showed that β-catenin is highly expressed in the cytoplasm and nucleus of melanoma-associated fibroblasts and is required for the biological functions of murine stromal fibroblasts.^2^ We initially discovered that B16 melanoma development was accelerated when β-catenin expression was ablated in melanoma-associated fibroblasts before tumor initiation.^10^ Surprisingly, in an in vivo study using a melanoma-CAF mouse model combining *Braf*^*V600E*^*; Pten*^*fl/fl*^ mouse melanoma cells^11,12^ with stromal fibroblasts of the genotype *Ctnnb1*^*fl/fl*^; *Col1α2-CreER*,^13^ we found that the growth of the *Braf*^*V600E*^*; Pten*^*fl/fl*^ melanoma was significantly suppressed upon β-catenin ablation in stromal fibroblasts following tumor formation, and this occurred through the downregulation of Erk/Mapk signaling.^14^ Despite the abundance of experimental evidence demonstrating the significance of β-catenin activity in CAFs, the molecular mechanisms underlying the functional association between β-catenin and the tumor-promoting and ECM remodeling abilities of CAFs have not been fully described.

In this study, we identified YAP as a direct β-catenin partner in stromal fibroblasts that modulates the biological activities of the cells. YAP has been previously shown to be a regulator of the differentiation of normal dermal fibroblasts into myofibroblasts, and it contributes to the maintenance of myofibroblast phenotypes.^15^ Our work uncovers a new role for the β-catenin-YAP signaling axis in melanoma-associated fibroblasts, wherein the axis regulates their stimulation and functions to promote ECM remodeling and cancer cell phenotypes.

## Results

### β-catenin contributes to the activation of stromal fibroblasts

**Fig. 1:**
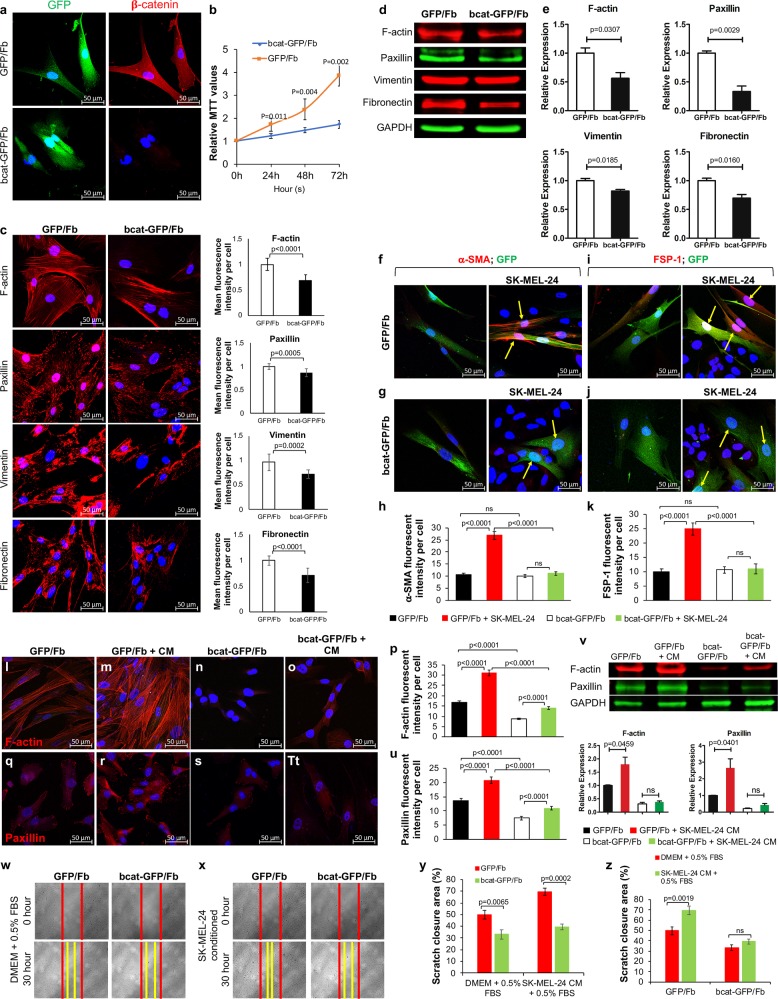
β-catenin is essential for the functional properties of stromal fibroblasts. **a** GFP/Fb and bcat-GFP/Fb were induced by addition of 500 ng/ml doxycycline to the culture medium for 72 h. Left: GFP expression in GFP/Fb and bcat-GFP/Fb; right: representative images of β-catenin immunofluorescence staining of GFP/Fb and bcat-GFP/Fb. Scale bar: 50 μm. **b** The viability of GFP/Fb and bcat-GFP/Fb was compared using the MTT assay. The cells were collected at 0, 24, 48, and 72 h. The data are representative of three independent experiments. **c** Expression of F-actin, paxillin, vimentin and fibronectin (red) in GFP/Fb and bcat-GFP/Fb was determined by immunostaining after a three-day doxycycline induction with DAPI counterstaining (blue). Scale bar: 50 μm. The mean fluorescence intensities of each immunostained protein in individual GFP/Fb and bcat-GFP/Fb cells were determined using ImageJ. **d** Expression of F-actin, paxillin, vimentin and fibronectin was measured in total protein extracted from 1 × 10^5^ GFP/Fb and bcat-GFP/Fb cells by Western blotting. GAPDH was used as an internal control. **e** The intensities of the bands of the indicated proteins expressed in GFP/Fb and bcat-GFP/Fb were normalized to the GAPDH signal. The data are representative of three independent experiments. **f**–**g** α-SMA immunofluorescence staining of GFP/Fb, GFP/Fb + SK-MEL-24 coculture, bcat-GFP/Fb, and bcat-GFP/Fb + SK-MEL-24 coculture with DAPI nuclear costaining. GFP/Fb and bcat-GFP/Fb were tagged with GFP and are denoted with yellow arrows in coculture images. Scale bar: 50 μm. **h** Fluorescence intensities of α-SMA expression were determined and compared between four groups from three individual experiments. **i–j** FSP-1 immunofluorescence staining of GFP/Fb, GFP/Fb + SK-MEL-24 coculture, bcat-GFP/Fb, and bcat-GFP/Fb + SK-MEL-24 coculture with DAPI nuclear costaining. **k** Fluorescence intensities of FSP-1 expression were determined and compared between four groups from three individual experiments. **l**–**o** F-actin immunofluorescent staining of GFP/Fb and bcat-GFP/Fb cultured in DMEM with 0.5% FBS and SK-MEL-24 CM with DAPI nuclear costaining. Scale bar: 50 μm. **q**–**t** Paxillin immunofluorescent staining of GFP/Fb and bcat-GFP/Fb cultured in DMEM with 0.5% FBS and SK-MEL-24 CM with DAPI nuclear costaining. Scale bar: 50 μm. **p** Fluorescence intensities of F-actin expression and **u**. paxillin were determined and compared between four groups from three individual experiments. **v** Expression of F-actin and paxillin was measured in total protein extracts from 1 × 10^5^ GFP/Fb and bcat-GFP/Fb cells cultured in DMEM with 0.5% FBS and SK-MEL-24 CM by Western blotting. GAPDH was used as an internal control. Statistical analysis of the intensities of the bands of indicated proteins expressed in GFP/Fb and bcat-GFP/Fb cultured in DMEM with 0.5% FBS and SK-MEL-24 CM were from three independent experiments and were normalized to the GAPDH band. **w** Representative images of the migration of GFP/Fb and bcat-GFP/Fb cultured in DMEM with 0.5% FBS at 0 and 30 h. Red lines indicate the starting edge of fibroblasts after the scratch. Yellow lines indicate the final distance that GFP/Fb and bcat-GFP/Fb migrated. **x** Representative images are shown of the migration of GFP/Fb and bcat-GFP/Fb cultured in SK-MEL-24 CM with 0.5% FBS at 0 and 30 h. Red lines indicate the starting points of fibroblasts after scratch. Yellow lines indicate the final distance that GFP/Fb and bcat-GFP/Fb migrated. The images shown are representative of at least three independently repeated experiments. **y** Statistical comparison was performed on the percentages of closed scratch areas between GFP/Fb and bcat-GFP/Fb under the same culture conditions. **z** Statistical comparison was performed on the percentages of closed scratch areas by GFP/Fb and bcat-GFP/Fb under different culture conditions.

The activation of the canonical WNT/β-catenin signaling pathway is associated with fibroblast activation, fibrosis, and tissue repair.^9,16,17^ We previously reported that CAFs infiltrating and surrounding human melanoma lesions express high levels of cytoplasmic and nuclear β-catenin.^10^ Further studies showed that targeted ablation of β-catenin in murine stromal fibroblasts had opposite biological effects on melanoma development depending on the timing of β-catenin ablation.^10,14^ Despite these interesting results, the mechanisms by which β-catenin regulates the biological properties of human stromal fibroblasts and their interactions with melanoma cells and the ECM remain largely unknown. To address this question, we used inducible lentiviral shRNAs (Fig. S1) to silence β-catenin expression in primary human dermal fibroblasts. Lentiviral vector uses an inducible Tet-On 3G bipartite gene silencing system and carry genes encoding both puromycin resistance and green fluorescence protein (GFP).^18^ Three different β-catenin-targeting shRNAs were designed (Fig. S1c) and evaluated for their abilities to inhibit β-catenin expression. bcat-GFP/Fb-3 shRNA was found to have the highest inhibitory efficiency (Fig. S1d-h) and was used to generate β-catenin-deficient stromal fibroblasts (hereafter referred to as bcat-GFP/Fb). Primary human fibroblasts transduced with a nontargeting shRNA were used as a control, and these cells were named as GFP/Fb. As shown in Fig. 1a, 72 h after doxycycline induction, the expression of β-catenin in bcat-GFP/Fb was significantly inhibited compared with that of GFP/Fb, while both GFP/Fb and bcat-GFP/Fb strongly expressed GFP. As expected, the number of viable bcat-GFP/Fb was always lower than that of GFP/Fb after the loss of β-catenin (Fig. 1b). This finding was consistent with our previous study, which showed that the loss of β-catenin in murine dermal fibroblasts caused cell cycle arrest and suppressed cell growth.^10^ In addition, as shown in Fig. 1c, bcat-GFP/Fb had decreased expression of the stress fiber F-actin, the focal adhesion protein paxillin, the class-III intermediate filament protein vimentin and the ECM protein fibronectin. Since the cell numbers were different between GFP/Fb and bcat-GFP/Fb after 72 h of culture, the mean fluorescence intensity of immunostained protein per cell in each group was quantified and compared. Bar graphs in Fig. 1c show that the loss of β-catenin led to reduced expression of respective proteins in stromal fibroblasts. Analysis of total proteins extracted from the same number of GFP/Fb and bcat-GFP/Fb cells using Western blotting confirmed that the overall expression of F-actin, paxillin, vimentin, and fibronectin was inhibited upon β-catenin ablation in stromal fibroblasts (Fig. 1d, e). These data suggest that β-catenin may contribute to the regulation of cytoskeletal organization and ECM production in stromal fibroblasts.

To determine whether β-catenin deficiency could inhibit human stromal fibroblasts from being activated by melanoma cells, we cocultured GFP/Fb and bcat-GFP/Fb with the melanoma cell line SK-MEL-24, which harbors a *BRAF*^*V600E*^ mutation.^19^ After a two-day coculture, the expression of α-smooth muscle actin (α-SMA) and fibroblast-specific protein 1 (FSP-1; also known as S100A4) was assessed as indicators of the activation state of fibroblasts by immunofluorescent staining.^20,21^ As shown in Fig. 1f–h, GFP/Fb cocultured with SK-MEL-24 melanoma cells exhibited upregulation of α-SMA expression in comparison to GFP/Fb in monoculture, which had very low expression of α-SMA. Interestingly, the expression of α-SMA in bcat-GFP/Fb was low in monoculture and did not significantly increase when cocultured with SK-MEL-24 cells (Fig. 1g, h). FSP-1 exhibited a very similar expression pattern as α-SMA (Fig. 1i–k). The findings showed that β-catenin is required in human stromal fibroblasts to respond to either direct or indirect signals from melanoma cells.

We next investigated the potential requirement of β-catenin in stromal fibroblasts to respond to indirect melanoma cell stimulation. Conditioned medium (CM) derived from SK-MEL-24 cells was collected to culture GFP/Fb and bcat-GFP/Fb. As shown in Fig. 1m, r, GFP/Fb had increased F-actin and paxillin expression when cultured in SK-MEL-24 CM compared with cells cultured in DMEM with 0.5% FBS (Fig. 1l, q; summarized in Fig. 1p, u). On the other hand, bcat-GFP/Fb had low expression of F-actin and paxillin (Fig. 1n, s) and did not respond to SK-MEL-24 CM (Fig. 1o, t) as GFP/Fb did. We also evaluated the expression of total F-actin and paxillin using the same number of GFP/Fb and bcat-GFP/Fb cultured in either DMEM with 0.5% FBS or SK-MEL-24 CM. As shown in Fig. 1v, the expression of F-actin and paxillin in GFP/Fb was significantly increased when cultured in SK-MEL-24 CM, while there was no significant change in bcat-GFP/Fb cultured in SK-MEL-24 CM.

The ability to migrate is one of the critical biological functions of stromal fibroblasts.^22^ Since the cytoskeleton and intracellular tension are important for the migratory ability of fibroblasts,^23^ we performed in vitro scratch assays to compare GFP/Fb and bcat-GFP/Fb cultured in DMEM with 0.5% FBS or SK-MEL-24 CM.^24^ As shown in Fig. 1w, y, when cultured in DMEM with 0.5% FBS, the percentage of the scratched area that was closed by bcat-GFP/Fb was 33.23 ± 4.08%, which was significantly less than that of GFP/Fb (49.74 ± 3.68%). Interestingly, as shown in Fig. 1z, the percentage of the scratched area that was closed by GFP/Fb cultured in SK-MEL-24 CM was increased to 69.44 ± 2.93% (left panel in Fig. 1x), which was greater than that of GFP/Fb cultured in DMEM with 0.5% FBS (left panel in Fig. 1w). However, bcat-GFP/Fb cultured in SK-MEL-24 CM (right panel in Fig. 1x) showed no significant improvement (39.40 ± 2.31%.) when compared to bcat-GFP/Fb cultured in DMEM with 0.5% FBS (right panel in Fig. 1w). These findings suggest that human stromal fibroblasts require β-catenin to respond to signals secreted by melanoma cells and to execute their biological functions.

### β-catenin is required by stromal fibroblasts to remodel the ECM

**Fig. 2:**
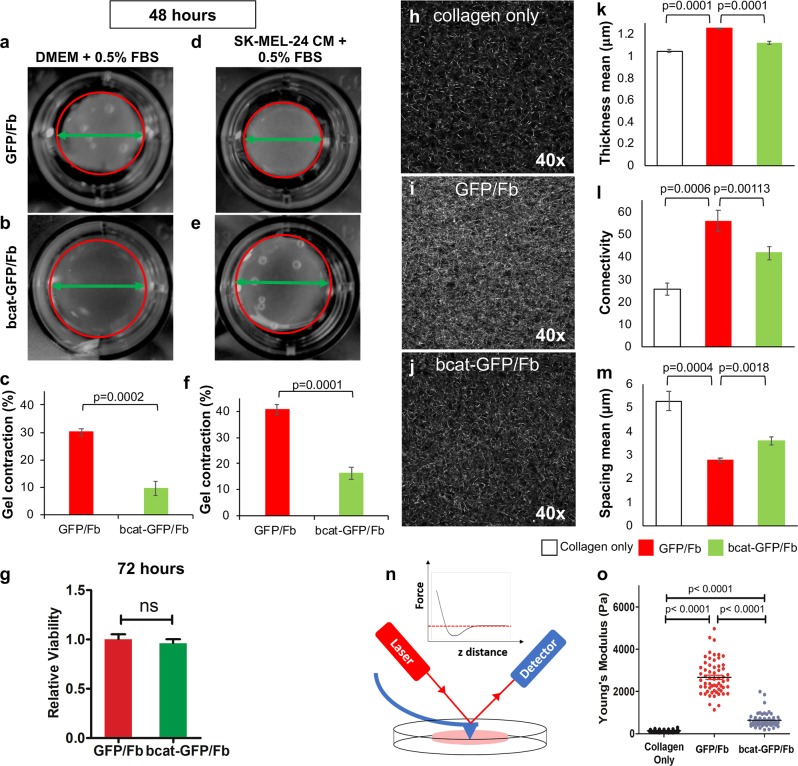
β-catenin is required by stromal fibroblasts to remodel the ECM. **a–d** Representative images of collagen gel contraction assays with GFP/Fb in either DMEM + 0.5% FBS **a** or SK-MEL-24 CM + 0.5% FBS **d** and with bcat-GFP/Fb in either DMEM + 0.5% FBS **b** or SK-MEL-24 CM + 0.5% FBS **e** after 48 h. The gel in each well is circled by a red line. The green double arrow line indicates the diameter of the contracted gel. **c** Statistical quantification is shown for the relative percentages of gel contraction by GFP/Fb and bcat-GFP/Fb cultured in DMEM + 0.5% FBS. **f** Statistical quantification is shown for the percentages of gel contraction by GFP/Fb and bcat-GFP/Fb cultured in SK-MEL-24 CM + 0.5% FBS. **g** Viability of GFP/Fb and bcat-GFP/Fb cultured in DMEM with 0.5% FBS after 72 h of culture was compared by the MTT assay. **h**–**j** CRM images of 1 mg/ml collagen gel alone **h**, gel with GFP/Fb **i** and gel with bcat-GFP/Fb **j** were acquired using a Zeiss LSM 510 Two-Photon microscope at 40X magnification three days after seeding. **k**–**m** CRM images were analyzed by ImageJ with the BoneJ plugin to compare fiber thickness **k**, connectivity **l** and spacing **m** among gels without cells, with GPF/Fb, and with bcat-GFP/Fb. **n** Illustration of the measurement of collagen gel stiffness using an atomic force microscope is shown. **o** Collagen gel stiffness was compared among collagen gel, collagen gel embedded with GFP/Fb and collagen gel embedded with bcat-GFP/Fb using AFM.

Collagen gel contraction assays were performed to evaluate the biological impact of β-catenin deficiency on the ECM remodeling ability of human stromal fibroblasts.^25^ As shown in Fig. 2c, after 48 h, the gel contracted by bcat-GFP/Fb in DMEM with 0.5% FBS was 9.60 ± 2.44% of the gel area (Fig. 2b), which was significantly less than that contracted by GFP/Fb (30.05 ± 1.68% in Fig. 2a). SK-MEL-24 CM enhanced the ability of GFP/Fb to contract collagen gels (40.58 ± 1.77% in Fig. 2d). However, the ability of bcat-GFP/Fb to contract collagen gel was only slightly enhanced by SK-MEL-24 CM (16.30 ± 2.24% in Fig. 2e), and it was still significantly less than that of GFP/Fb (Fig. 2f). The difference in collagen gel contractions between GFP/Fb and bcat-GFP/Fb was not due to a difference in cell proliferation. As shown in Fig. 2g, the viability of bcat-GFP/Fb was comparable to that of GFP/Fb when cultured in DMEM with 0.5% FBS after 72 h. Thus, β-catenin is required by stromal fibroblasts to perform its ECM remodeling functions and to respond to the stimulatory signals released by cancer cells.

We next visualized and quantified collagen fiber alignment and reorganization using confocal reflection microscopy (CRM).^26^ As shown in Fig. 2i, a more densely populated fiber content was observed in collagen gel embedded with GFP/Fb in comparison to what was observed in the collagen gel-only sample (Fig. 2h), suggesting that embedded fibroblasts contributed to collagen deposition and reorganization. However, after ablating β-catenin in stromal fibroblasts, the density of collagen fibers in the gel was only slightly increased (Fig. 2j) compared with the collagen gel only sample and significantly less than that of collagen gel embedded with GFP/Fb. ImageJ with the BoneJ plugin was used to analyze CRM image data and compare the matrix organization. The analysis showed increased fiber thickness (Fig. 2k), increased connectivity (Fig. 2l), and decreased fiber spacing (Fig. 2m) in collagen gels embedded with GFP/Fb compared to that of the collagen gel only controls. Connectivity describes the number of connected structures in a network, revealing the complexity of collagen fibers in the gel.^27^ Both fiber thickness and connectivity were reduced, while fiber spacing was increased in collagen gels after β-catenin expression was inhibited in stromal fibroblasts. The CRM data correlated well with the results obtained from the collagen gel contraction assays, highlighting the importance of β-catenin for the ability of stromal fibroblasts to remodel the ECM. The stiffness of collagen gels was measured using an atomic force microscope (AFM), as depicted in Fig. 2n. The stiffness of the gel embedded with GFP/Fb increased more than tenfold over that of the collagen only control (Fig. 2o). However, after β-catenin expression was silenced in fibroblasts, the stiffness of the matrix was only slightly increased over the collagen-only control and significantly less than that of the gel embedded with GFP/Fb, further demonstrating that β-catenin is a critical factor for fibroblasts to remodel the ECM.

### Stromal β-catenin contributes to the growth of BRAF-mutant melanoma cells

**Fig. 3:**
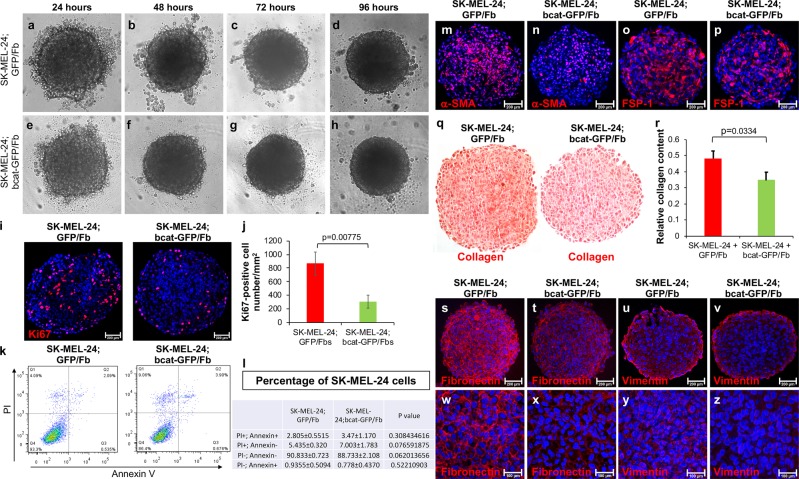
Stromal fibroblasts require β-catenin to support melanoma cell growth. SK-MEL-24; GFP/Fb spheroid and SK-MEL-24; bcat-GFP/Fb spheroid cultures were grown for 96 h. Representative bright-field images of SK-MEL-24; GFP/Fb spheroid **a**–**d** and SK-MEL-24; bcat-GFP/Fb spheroid cultures **e**–**h** were taken using a Carl Zeiss Axiovert 100 TV inverted microscope at ×5 magnification at 24, 48, 72, and 96 h. **i** Ki67 immunofluorescence staining (red) of SK-MEL-24; GFP/Fb and SK-MEL-24; bcat-GFP/Fb spheroids embedded in paraffin. The nuclei were stained with DAPI (blue). Scale bar: 200 μm. **j** Quantification of Ki67-positive cells per square millimeter of SK-MEL-24; GFP/Fb spheroid sections and SK-MEL-24; bcat-GFP/Fb spheroid sections. A minimum of ten randomly selected fields was counted for each spheroid type. **k** Flow cytometry was used to analyze apoptosis and death of SK-MEL-24 melanoma cells in both SK-MEL-24; GFP/Fb spheroids and SK-MEL-24; bcat-GFP/Fb spheroids using annexin V and PI staining. **l** Percentages of SK-MEL-24 cell subpopulations based on PI and annexin V staining in SK-MEL-24; GFP/Fb spheroids and SK-MEL-24; bcat-GFP/Fb spheroids are shown. **m**–**p** α-SMA **m–n** and FSP-1 **o**–**p** immunostaining was performed on 5-μm-thick paraffin sections of SK-MEL-24; GFP/Fb and SK-MEL-24; bcat-GFP/Fb spheroids. Pictures were taken using a Nikon microscope at ×20 magnification. Scale bar: 200 μm. **q** Collagen content in SK-MEL-24; GFP/Fb spheroids and SK-MEL-24; bcat-GFP/Fb spheroids was visualized by picrosirius red staining. Pictures were taken using an inverted light microscope at ×20 magnification. **r** Quantification of collagen content was performed by collagen extraction and colorimetric measurement. Collagen content was normalized to total protein content for each sample. **s**–**v** Fibronectin **s**–**t** and vimentin **u**–**v** immunostaining was performed on 5-μm-thick paraffin sections of SK-MEL-24; GFP/Fb and SK-MEL-24; bcat-GFP/Fb spheroids. Pictures were taken using a Nikon microscope at ×20 magnification. Scale bar: 200 μm. **w**–**z** Photographs were taken at ×40 magnification. Scale bar: 100 μm.

To assess whether the loss of β-catenin in stromal fibroblasts would have any negative effects on *BRAF*^*V600E*^ human melanoma cell growth, we cocultured SK-MEL-24 melanoma cells with either GFP/Fb (Fig. 3a–d) or bcat-GFP/Fb (Fig. 3e–h) in three-dimensional (3D) spheroids for 96 h. Because no significant difference can be seen between the morphology of cocultured spheroids, including the sizes, we decided to evaluate the expression of cell proliferation and apoptosis markers. Ki67 is a nuclear antigen expressed in mid-G1, S, G2 and M phases of the cell cycle, and it serves as a marker of cell proliferation.^28^ The expression of Ki67 was significantly reduced in SK-MEL-24; bcat-GFP/Fb spheroids compared with SK-MEL-24; GFP/Fb spheroids (Fig. 3i). As shown in Fig. 3j, there was an average of 868 ± 172 Ki67-positive cells per square millimeter section of SK-MEL-24; GFP/Fb spheroids, while there were only 305 ± 95 Ki67-positive cells per square millimeter section of SK-MEL-24; bcat-GFP/Fb spheroids. To determine whether β-catenin deficiency in stromal fibroblasts would cause increased melanoma cell death and apoptosis in cocultured spheroids, annexin V-APC and propidium iodide (PI) staining was performed for flow cytometry analysis.^29^ Because stromal fibroblasts were labeled with GFP, we were able to identify melanoma cells by flow cytometry and calculated the percentage of melanoma cells that were dead or apoptotic. Surprisingly, only a slight increase in cell death and apoptosis occurred in SK-MEL-24 melanoma cells in SK-MEL-24; bcat-GFP/Fb spheroids as compared with those SK-MEL-24; GFP/Fb spheroids (Fig. 3k). As shown in Fig. 3l, there were 2.81 ± 0.55% PI-positive; Annexin V-positive melanoma cells and 5.44 ± 0.32% PI-positive; Annexin V-negative melanoma cells in SK-MEL-24; GFP/Fb spheroids; however, there were 3.47 ± 1.17% PI-positive; Annexin V-positive melanoma cells and 7.00 ± 1.78% PI-positive; Annexin V-negative melanoma cells in SK-MEL-24; bcat-GFP/Fb spheroids. The above data suggest that β-catenin deficiency in stromal fibroblasts mainly has negative effects on melanoma cell growth in spheroids and does not lead to significant melanoma cell death.

We evaluated the expression of two known CAF markers, α-SMA and FSP-1, in both types of spheroid. As shown in Fig. 3m, n, the number of cells expressing α-SMA was 532 ± 94 per square millimeter section of SK-MEL-24; GFP/Fb spheroids, while the number of α-SMA-positive cells per square millimeter section of SK-MEL-24, bcat-GFP/Fb spheroids was 117 ± 91. There were 180 ± 50 FSP-1-positive cells per square millimeter section in SK-MEL-24, bcat-GFP/Fb spheroids (Fig. 3p), while in SK-MEL-24, GFP/Fb spheroid sections, the number of FSP-1-positive cells was 464 ± 72 (Fig. 3o). The data suggest that the number of activated stromal fibroblasts in spheroids was reduced due to β-catenin deficiency. Activated stromal fibroblasts are considered to be the major producer of collagen and ECM proteins in the tumor stroma, which is known for influencing cancer cell behaviors.^30^ Therefore, we assessed the collagen content in spheroids formed by SK-MEL-24 with GFP/Fb or bcat-GFP/Fb by picrosirius red staining.^31^ As shown in Fig. 3q, the SK-MEL-24; bcat-GFP/Fb spheroids had less collagen content than the control SK-MEL-24; GFP/Fb spheroids. Quantification of collagen content confirmed that there was a 28.01% reduction in collagen in SK-MEL-24; bcat-GFP/Fb spheroids (Fig. 3r).^32^ The expression of fibronectin and vimentin was also decreased in SK-MEL-24; bcat-GFP/Fb spheroids (Fig. 3t, v) when compared with SK-MEL-24; GFP/Fb spheroids (Fig. 3s, u). It appeared that SK-MEL-24; GFP/Fb spheroids had much denser networks of fibronectin and vimentin (Fig. 3w, y) than the SK-MEL-24; bcat-GFP/Fb spheroids (Fig. 3x, z). This observation confirmed our previous observation that β-catenin is required for biological functions of stromal fibroblasts.

To ensure that the data were reproducible, we repeated the 3D spheroid coculture experiments using a red fluorescent protein (RFP)-tagged melanoma cell line, A375, so that melanoma cells and stromal fibroblasts could be easily distinguished based on their fluorescence. As shown in Fig. S2a and S2d, spheroids formed by A375 and stromal fibroblasts displayed a growth pattern that was similar to those formed by SK-MEL-24 and stromal fibroblasts. However, there were clear differences in the growth of A375 (in red, Fig. S2b vs. S2e) and stromal fibroblasts (in green fluorescence; Fig. S2c vs. S2f) after β-catenin was ablated in the fibroblasts. We quantified the mean fluorescent intensities of A375 melanoma cells (red) in each spheroid after a 144-hour culture. As shown in Fig. S2g, A375 and GFP/Fb cocultures had higher red fluorescence than A375 and bcat-GFP/Fb cocultures. The differences were validated by quantifying the number of RFP-tagged A375 cells in individual spheroids using a Countess II Automated Cell Counter (Fig. S2h).

### Nuclear YAP translocation is inhibited in melanoma-associated fibroblasts after β-catenin ablation

**Fig. 4:**
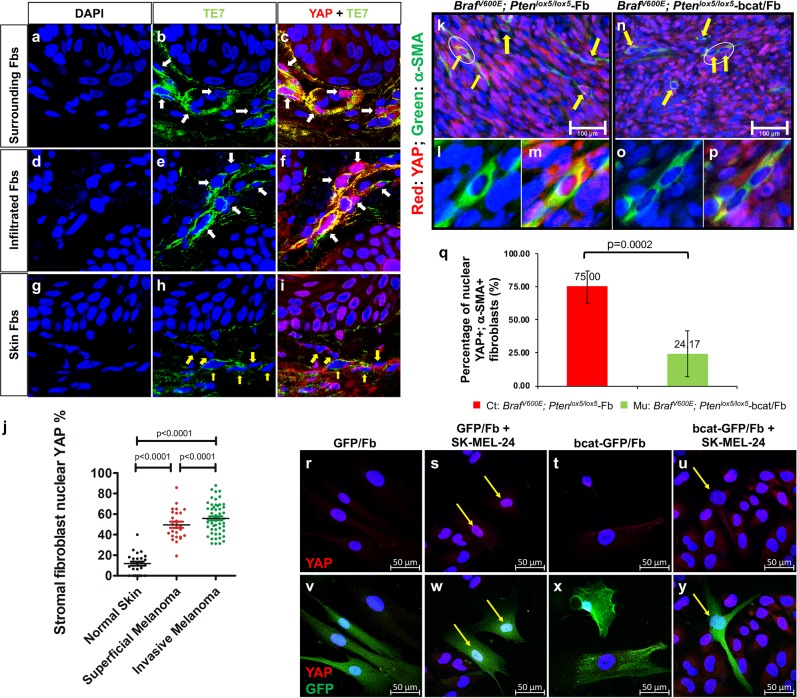
The nuclear translocation of YAP is inhibited in melanoma-associated fibroblasts after β-catenin ablation. The expression of nuclear YAP (red) in fibroblasts (Fbs) in human melanoma tissue (indicated by white arrows) was visualized by coimmunostaining with a fibroblast-specific anti-TE7 antibody (green). **a**–**c** Surrounding Fbs. **d**–**f** Infiltrated Fbs. **g**–**i** Human skin Fbs. Images shown are typical of ten human melanoma samples for each type. **j** Comparison of the percentages of stromal fibroblasts expressing nuclear YAP among the following: fibroblasts surrounding melanoma, infiltrated fibroblasts in melanoma and stromal fibroblasts in normal human skin. The percentage of stromal fibroblasts expressing nuclear YAP was calculated by dividing the number of nuclear YAP-positive and TE7-positive cells by the total number of TE7-positive cell number in each group. Statistical analysis of differences was carried out by Student’s *t*-tests using GraphPad Prism. **k** Stromal fibroblasts in *Braf*^*V600E*^*; Pten*^*lox5*^-Fb tumors express cytoplasmic and nuclear YAP. Yellow arrows point to fibroblasts expressing nuclear YAP. **l**–**m** Enlarged images are shown of fibroblasts circled in **k** with single α-SMA staining **l** and double YAP/α-SMA staining **m**. **n** In *Braf*^*V600E*^*; Pten*^*lox5*^-bcat/Fb tumors, not only is the number of activated fibroblasts reduced but also the nuclear translocation of YAP was inhibited in fibroblasts. **o**–**p** Enlarged images are shown of fibroblasts circled in D with single α-SMA staining **o** and double YAP/α-SMA staining **p** The data are representative of three independent experiments. Scale bar: 100 μm. **q** The percentage of α-SMA-positive cells expressing nuclear YAP in *Braf*^*V600E*^*; Pten*^*lox5*^-Fb tumors and *Braf*^*V600E*^*; Pten*^*lox5*^-bcat/Fb tumors is shown. Five different pairs of *Braf*^*V600E*^*, Pten*^*lox5*^-Fb tumors and *Braf*^*V600E*^*, and Pten*^*lox5*^-bcat/Fb tumors were stained and counted. **r**–**y** YAP immunofluorescence staining of GFP/Fb, GFP/Fb + SK-MEL-24 coculture, bcat-GFP/Fb, and bcat-GFP/Fb + SK-MEL-24 coculture with DAPI nuclear costaining (blue). GFP/Fb and bcat-GFP/Fb were tagged with GFP and are denoted with yellow arrows in coculture pictures. Scale bar: 50 μm.

It was reported that there is a crosstalk between YAP and the Wnt/β-catenin signaling pathway.^33–35^ To assess the relevance of YAP nuclear translocation in fibroblast activation, we costained normal human skin and melanoma tissue paraffin sections with an anti-YAP antibody and a specific anti-fibroblast antibody, TE7.^36^ As shown in Fig. 4c, f, surrounding and infiltrated fibroblasts in invasive melanoma lesions expressed a high level of nuclear YAP, while a low level of nuclear YAP was observed in normal human dermal fibroblasts (Fig. 4i), suggesting that activated YAP is potentially involved in the biological activities of melanoma-associated fibroblasts. As shown in Fig. 4j, an average of 49.6 ± 14.42% of TE7-positive fibroblasts in superficial melanoma expressed nuclear YAP, and 55.8 ± 14.46% of TE7-positive fibroblasts in invasive melanoma expressed nuclear YAP; however, only 11.7 ± 9.92% dermal fibroblasts showed nuclear translocation of YAP, suggesting that YAP plays an important role in melanoma-associated fibroblasts.

To understand whether β-catenin is related to YAP nuclear localization in melanoma-associated fibroblasts, we collected tumor tissues from mice bearing *Braf*^*V600E*^*; Pten*^*lox5*^*-*Fb or *Braf*^*V600E*^*; Pten*^*lox5*^-bcat/Fb melanoma^14^ for double staining using an anti-YAP antibody and an anti-α-SMA antibody. A majority of α-SMA-positive fibroblasts in control *Braf*^*V600E*^*; Pten*^*lox5*^*-*Fb melanomas showed red fluorescent staining of YAP (Fig. 4m) in the DAPI-counterstained blue nuclei (Fig. 4l), indicating the nuclear expression of YAP (indicated by yellow arrows in Fig. 4k). However, there was a clear loss of nuclear YAP in β-catenin-deficient α-SMA-positive fibroblasts in *Braf*^*V600E*^*; Pten*^*lox5*^-bcat/Fb melanomas (Fig. 4n). As shown in Fig. 4p, no red fluorescent YAP staining was observed in the blue nucleus (Fig. 4o). Cell counting revealed that 75 ± 12.2% of α-SMA-positive fibroblasts in control melanomas expressed nuclear YAP, while only 24.17 ± 17.4% α-SMA-positive fibroblasts in *Braf*^*V600E*^*; Pten*^*lox5*^-bcat/Fb melanomas possessed nuclear YAP (Fig. 4q). The contribution of β-catenin to YAP nuclear translocation was also confirmed by coculture experiments. As shown in Fig. 4s, GFP/Fb cocultured with SK-MEL-24 melanoma cells showed upregulation of nuclear YAP over what was observed with GFP/Fb alone (Fig. 4r). Interestingly, the expression and nuclear localization of YAP in bcat-GFP/Fb were not significantly increased when cocultured with SK-MEL-24 cells (Fig. 4u); the expression and localization were comparable to those of bcat-GFP/Fb (Fig. 4t).

### YAP physically interacts with β-catenin in stromal fibroblasts

**Fig. 5:**
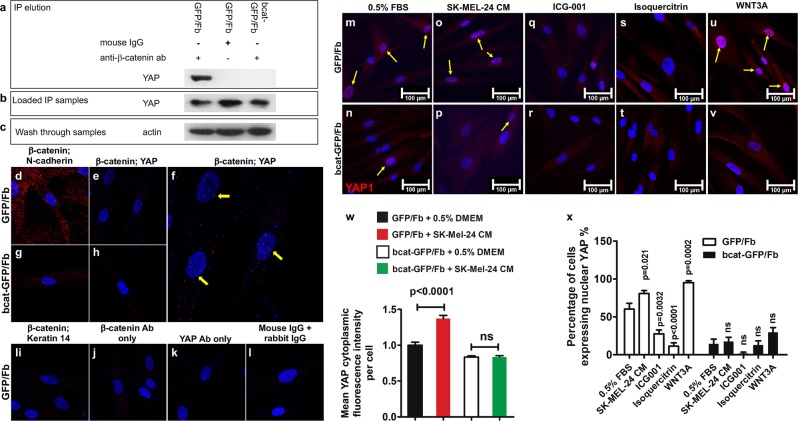
β-catenin interacts with YAP in human stromal fibroblasts. **a**–**c** Coimmunoprecipitation analysis. Total GFP/Fb and bcat-GFP/FB protein lysates were subjected to immunoprecipitation (IP) using the indicated antibodies: anti-β-catenin antibody and mouse IgG. **a** Immuno-complexes were analyzed by Western blotting with an anti-YAP antibody. **b** The middle panel shows the presence of YAP protein in cell lysates. **c**. Actin expression in the column wash through from IP. **d**–**l** PLA was performed to confirm the YAP and β-catenin interaction. GFP/Fb and bcat-GFP/Fb were seeded in chamber slides for PLA. A clear YAP-β-catenin interaction can be seen in GFP/Fb **e** but not bcat-GFP/Fb **h**. **f** Nuclear YAP-β-catenin interaction can be seen in the nuclei of GFP/Fb. N-cadherin was used as a positive control **d**, **g**, and keratin 14 was used as a negative control **i**. No signal could be detected in the β-catenin antibody-only treatment **j**, YAP antibody only treatment **k** and mouse IgG + rabbit IgG treatment **l**. **m**–**v** Wnt signaling regulates YAP nuclear translocation. GFP/Fb and bcat-GFP/Fb were seeded in chamber slides and treated with DMEM with 0.5% FBS **m**, **n**, SK-MEL-24 CM **o**, **p**, 100 μM ICG-00 **q**, **r**, 100 μM isoquercitrin **s**, **t** and 100 ng/ml WNT3A **u**, **v**. Yellow arrows indicate the cells expressing nuclear YAP. The pictures shown are representative of three independent experiments. **w** The mean fluorescence intensities of cytoplasmic YAP in individual GFP/Fb and bcat-GFP/Fb when cultured in DMEM with 0.5% FBS and SK-MEL-24 CM were determined using ImageJ and were normalized according to the intensity of cytoplasmic YAP in GFP/Fb cultured in DMEM with 0.5% FBS. A minimum of thirty cells in each group/culture condition were randomly selected from three independent experiments and were quantified. **x** Percentages of GFP/Fb and bcat-GFP/Fb expressing nuclear YAP were calculated by counting and dividing the number of nuclear YAP-positive cells by the total cell number under the following conditions: including DMEM with 0.5% FBS, SK-MEL-24 CM, 100 μM ICG-00, 100 μM isoquercitrin and 100 ng/ml WNT3A. At least fifty cells in each group/culture condition were randomly selected from three independent experiments and were counted.

We performed coimmunoprecipitation to determine whether YAP and β-catenin interact with each other in stromal fibroblasts. As shown in Fig. 5a, a YAP band could be detected by Western blotting in the eluate that was coprecipitated with an anti-β-catenin antibody from the GFP/Fb lysate. However, no YAP was coimmunoprecipitated from GFP/Fb lysate using a mouse IgG control antibody or from bcat-GFP/Fb lysate using an anti-β-catenin antibody. YAP protein was present in all cell lysates before incubation with the anti-β-catenin antibody (Fig. 5b). To further validate direct YAP-β-catenin interactions, we performed proximity ligation assays (PLA) using GFP/Fb and bcat-GFP/Fb. N-cadherin is a known β-catenin-interacting protein and was used as a positive control, while keratin 14 was used as a negative control. As shown in Fig. 5d, there was a strong interaction between β-catenin and N-cadherin in GFP/Fb, which was indicated by red fluorescent dots. However, this interaction was dramatically reduced in bcat-GFP/Fb due to β-catenin ablation (Fig. 5g). β-catenin and YAP interactions could be detected in GFP/Fb (Fig. 5e), which included a potential signal in the nucleus (Fig. 5f), but there was no signal in the bcat-GFP/Fb (Fig. 5h). As the cells were grown on a slide, we cannot exclude the possibility that the positive β-catenin and YAP interaction shown in the nuclear area was actually in the cytoplasm that overlapped with the nucleus. No interaction between β-catenin and keratin 14 was detected (Fig. 5i).

Cultured GFP/Fb and bcat-GFP/Fb were treated with either Wnt inhibitors or Wnt ligands to determine whether Wnt/β-catenin signaling regulates YAP nuclear localization (Fig. 5x). YAP protein could be readily identified in GFP/Fb (Fig. 5m) but was only weakly detected in bcat-GFP/Fb (Fig. 5n) that were cultured in DMEM with 0.5% FBS. When the cells were treated with SK-MEL-24 CM, the number of GFP/Fb expressing nuclear YAP was significantly increased (Fig. 5o), but there was only a slight increase in the number of bcat-GFP/Fb expressing nuclear YAP (Fig. 5p). In addition, as shown in Fig. 5w, cytoplasmic YAP was increased in GFP/Fb (Fig. 5o), but there was no increase in YAP expression in bcat-GFP/Fb (Fig. 5p). Two WNT inhibitors, ICG-100 and isoquercitrin, were used to inhibit the Wnt/β-catenin signaling pathway. ICG-100 is an inhibitor of the CREB-binding protein,^37^ which is known to interact with β-catenin to promote tumor cell growth.^38^ Isoquercitrin inhibits Wnt/β-catenin signaling by acting downstream of β-catenin translocation to the nucleus.^39^ As shown in Fig. 5q, s, the nuclear expression of YAP in GFP/Fb was significantly reduced in response to these inhibitors. The numbers of bcat-GFP/Fb expressing nuclear YAP were also further decreased when the cells were treated with ICG-100 and isoquercitrin (Fig. 5r, t). However, when the cells were treated with WNT3A to activate Wnt signaling, the number of GFP/Fb expressing nuclear YAP was highly increased (Fig. 5u), but the same effect was not observed in bcat-GFP/Fb (Fig. 5v).

### YAP is essential for the ECM remodeling function of stromal fibroblasts

**Fig. 6:**
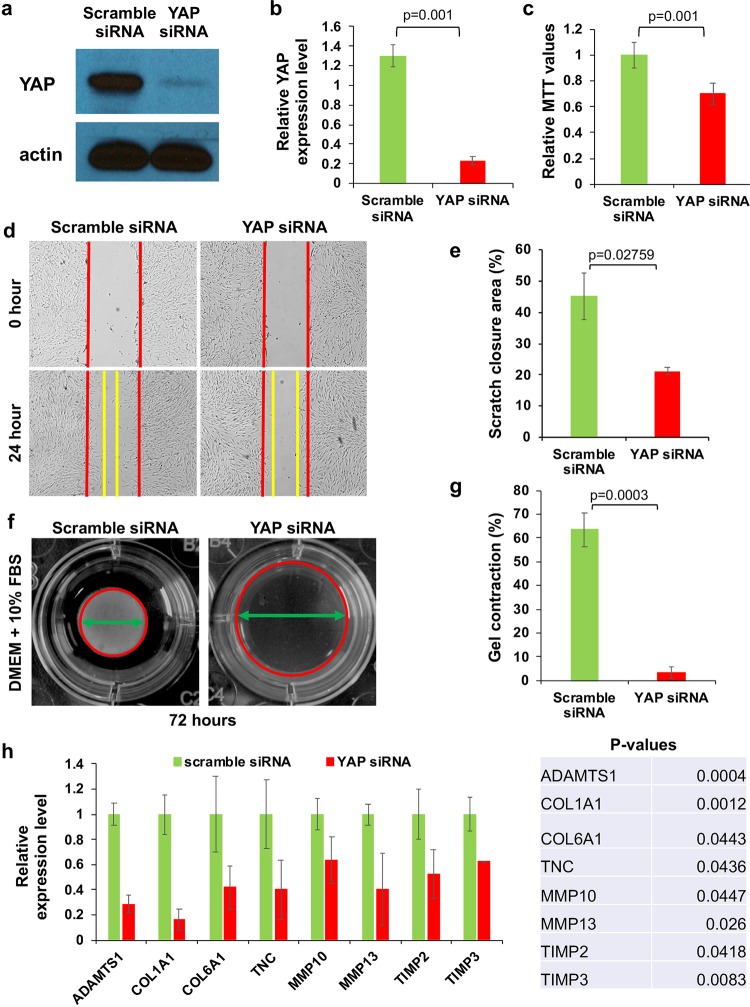
YAP is essential for the biological functions of fibroblasts. **a** YAP expression in fibroblasts transfected with YAP siRNA and scrambled siRNA is shown by Western blotting. **b** YAP expression was quantified and normalized to the internal control, actin**. c** The viability of YAP siRNA-transfected fibroblasts and control fibroblasts was compared using the MTT assay. **d** Migration of YAP siRNA-transfected and scramble siRNA-transfected fibroblasts was compared by the scratch assay. **e** Statistical comparison was performed for the percentages of closed scratch areas by YAP siRNA-transfected and scramble siRNA-transfected fibroblasts from three independent experiments. **f** Gel contraction assays were used to compare the ECM remodeling abilities of YAP siRNA-transfected fibroblasts and control fibroblasts at 72 h. **g** Statistical quantification of the relative percentages of gel contraction by human stromal fibroblasts transfected with YAP siRNA and scramble siRNA. **h** The production of ECM proteins and enzymes by YAP siRNA-transfected fibroblasts and control fibroblasts after 72 h of culture was quantified by qPCR and was statistically analyzed using Prism.

To understand the biological role of YAP in stromal fibroblasts, we silenced YAP expression in human stromal fibroblasts using a small interfering RNA (siRNA). As shown in Fig. 6a, YAP expression was significantly reduced in fibroblasts transfected with a YAP siRNA when compared to control fibroblasts transfected with a scramble siRNA. We quantified the expression of YAP and normalized it to the internal control β-actin, and we found an 83% reduction of YAP expression in YAP siRNA-transfected fibroblasts compared to YAP levels in fibroblasts transfected with scramble siRNA (Fig. 6b). Next, we evaluated the ability of fibroblasts to grow and migrate after YAP silencing. As shown in Fig. 6c, the growth of fibroblasts was suppressed after YAP expression was silenced. The in vitro scratch assay revealed that fibroblasts transfected with YAP siRNA migrated much slower than control fibroblasts (Fig. 6d). As shown in Fig. 6e, the percentage of the scratched area that was closed by fibroblasts transfected with YAP siRNA was 21.19 ± 1.26%, which was significantly less than the area closed by fibroblasts transfected with scramble siRNA (45.74 ± 7.41%).

We next performed collagen gel contraction assays to evaluate the role of YAP in ECM remodeling. After a 24-h incubation, both gels were detached from the bottom of the wells for contraction. As shown in Fig. 6f, g, after 72 h, fibroblasts transfected with scramble siRNA contracted approximately 63.46 ± 7.11% of the gel area. In contrast, collagen gels embedded with YAP siRNA-transfected fibroblasts only contracted 3.25 ± 2.33% of the gel area. To determine whether the inhibition of YAP expression suppressed the expression of ECM proteins and enzymes required for remodeling of the ECM, both types of fibroblasts were cultured for 72 h and were then collected for quantification of gene expression by real-time PCR. As shown in Fig. 6h, the mRNA expression of a variety of ECM proteins and MMPs was significantly reduced, supporting the notion that the matrix-remodeling ability of stromal fibroblasts was impaired due to YAP deficiency.

## Discussion

Melanomas are a solid tumor type comprising two discrete but interdependent cell compartments: melanoma cells and stromal cells.^40^ Unlike melanoma cells, stromal cells, including endothelial cells, immune cells, and fibroblasts, are genetically stable and are not the source of the disease.^41^ However, they do play important roles in creating a pro-tumor microenvironment and shaping the complexity of the TME. Among tumor-associated stromal cells, CAFs constitute the bulk of solid tumor stroma and contribute to tumor progression and therapeutic response.^42^ Furthermore, tumor cells are known to be influenced by the composition, organization and mechanical properties of the microenvironment as well as by molecular signals released by stromal cells.^43^ In this regard, CAFs are important because they synthesize the majority of ECM proteins, including fibrillar collagens and fibronectin, to promote matrix stiffening. On the other hand, ECM remodeling requires the presence of ECM-degrading enzymes, such as matrix metalloproteinases (MMPs), and collagen cross-linking enzymes, such as lysyl oxidase. CAFs are known to produce and secrete those types of ECM enzymes, which facilitate matrix reorganization and subsequently the motility and invasiveness of melanoma cells.^44^ Moreover, it was reported that increased ECM stiffness drives cytoskeletal tension in CAFs by modulating cellular actomyosin contractility, promoting a positive feedback loop between CAF functions and matrix properties, which is critical for tumor phenotypes.^45^ While these discoveries shed light on the contributions of CAFs and matrix remodeling to tumor progression, the molecular mechanisms underlying the interplay among stromal fibroblasts, melanoma cells and the ECM remain to be further elucidated.

As a dual functional protein, β-catenin expression is highly upregulated in both the nuclei and cytoplasm of CAFs in human melanoma stroma compared with normal dermal fibroblasts,^10^ suggesting that it might be important for the biological properties of melanoma-associated fibroblasts. In our study, human stromal fibroblasts showed decreased viability after the loss of β-catenin. This observation was expected because β-catenin is known to be the key mediator of Wnt signaling that regulates the expression of genes needed for cell proliferation and differentiation.^46^ Another key finding is that fibroblasts exhibited cytoskeletal changes after β-catenin ablation, such as reduced levels of the stress fiber F-actin, the intermediate filament vimentin, and the focal adhesion molecule paxillin. This deficiency could be because β-catenin has a significant role in maintaining and remodeling cytoskeletal structures in stromal fibroblasts, as it is known that β-catenin interacts with α-catenin and the actin cytoskeleton.^47^ Another possibility is that the expression of specific genes that are needed for cytoskeletal maintenance was impaired due to β-catenin ablation. However, these changes could also be secondary effects resulting from the inhibition of stromal fibroblast proliferation after β-catenin ablation. To conclude, the exact reasons that lead to cytoskeletal changes in β-catenin-deficient fibroblasts remain to be elucidated.

Although it is known that stromal fibroblasts are stimulated by melanoma cells to enter a state of activation and subsequently establish a microenvironment that is conducive for melanoma development, the underlying mechanisms remain poorly understood. The first question that we wanted to address was whether β-catenin is required for human stromal fibroblasts to respond to melanoma stimulation. Therefore, we cocultured human stromal fibroblasts with SK-MEL-24 melanoma cells so that both direct and indirect contacts could be established between the two types of cells. α-SMA and FSP-1 are known markers expressed in CAFs, and both were greatly decreased in β-catenin-deficient human fibroblasts. Their reduced expression suggested that stromal fibroblasts failed to respond to stimulation derived from human melanoma cells upon the ablation of β-catenin expression. To further understand whether melanoma cells could indirectly stimulate stromal fibroblasts and whether β-catenin is needed for this stimulatory effect, we evaluated the migratory ability of stromal fibroblasts when the cells were stimulated by culture medium conditioned by SK-MEL-24 cells. Ablation of β-catenin in human stromal fibroblasts significantly decreased their ability to migrate and respond to indirect melanoma stimulation. The phenotype was further confirmed by the finding that bcat-GFP/Fb failed to increase the expression of F-actin and focal adhesion protein paxillin upon stimulation with conditioned medium.

Fibroblasts grown in collagen gel are capable of causing gel contraction by rearranging the ECM and reorienting collagen fibers.^48^ In particular, when fibroblasts are activated by tumor cells to transdifferentiate and express a myofibroblast phenotype, their ability to induce gel contraction increases.^49^ Our study showed that β-catenin plays an important role in the ability of stromal fibroblasts to contract collagen gels. Furthermore, although the medium conditioned by SK-MEL-24 cells increased this myofibroblast-like phenotype in control fibroblasts, β-catenin-deficient stromal fibroblasts were not able to respond similarly; they remained unable to remodel the collagen matrix. This observation is consistent with our scratch assay data that show the response of stromal fibroblasts to signals released by tumor cells is largely dependent on β-catenin. CRM and AFM data also demonstrated that β-catenin activity is required for stromal fibroblasts to remodel and stiffen the matrix.

Increased tissue remodeling and ECM stiffness in the tumor stroma promote cell signaling pathways that are important for melanoma cells to grow, survive, migrate, and undergo epithelial-mesenchymal transition (EMT).^50^ The results obtained from the cocultures of stromal fibroblasts with either SK-MEL-24 or A375 melanoma cells clearly reinforced this idea. Both melanoma cell lines lacked robust growth after the loss of β-catenin in fibroblasts. One possible reason for the growth reduction is the lack of appropriate remodeling of the ECM microenvironment due to the changes in stromal fibroblasts after β-catenin ablation. Indeed, we found decreased collagen, fibronectin and vimentin content in the cocultured spheroid stroma, suggesting that there were changes in ECM dynamics and organization that influenced cellular behaviors of melanoma cells. These deficiencies could be because β-catenin interacts with α-catenin or focal adhesion proteins to connect the actin cytoskeleton with membrane proteins, such as N-cadherin or integrin receptors, which subsequently establishes contacts between stromal fibroblasts and other cell types or ECM components within the stroma. These cell-cell and cell-ECM contacts may act as a way to reciprocate the environmental cues with the biological activity of stromal fibroblasts.^51^ When β-catenin is lost in stromal fibroblasts, cell-cell and cell-ECM interactions are interrupted in fibroblasts. These changes have negative impacts on stromal fibroblasts, leading to decreased production of ECM components, e.g., collagens, fibronectin, and ECM enzymes, such as MMPs.

In addition to its adhesive functions, β-catenin also serves as a central factor in canonical Wnt signaling and can interact with a range of transcription factors in the nucleus. YAP is one of these factors. YAP is a downstream transcription coactivator governed by a cascade of kinases in the Hippo signaling pathway.^52^ This cascade involves a series of protein serine kinases, including mammalian sterile twenty-like kinases 1/2 (MST1/2, Hippo homologs; also known as STK3/K4) and large tumor suppressor kinases 1/2 (LATS1/2, Warts homologs).^53^ The Hippo signaling pathway is considered to be an important tumor-suppressor pathway, and its dysregulation has been noted in a variety of human cancers in which YAP nuclear translocation activates target gene transcription and enables cancerous cells to overcome contact inhibition and to grow and spread uncontrollably.^54^ Furthermore, YAP activity is believed to be important for fibroblast activation, proliferation, and differentiation. YAP has been shown to mediate the differentiation of dermal fibroblasts into myofibroblasts, and it contributes to the maintenance of myofibroblast phenotypes.^15^ Lee *et al*. reported that YAP expression was highly increased in dermal fibroblasts in wounds, and YAP knockdown significantly delayed the wound-healing process.^55^ CAFs in solid tumors have characteristics similar to those of myofibroblasts and wound fibroblasts.^56^ Calvo *et al*. reported that nuclear YAP activity is required to maintain CAF tumor-promoting phenotypes, including matrix stiffening, cancer cell invasion, and angiogenesis.^45^ It is believed that the reciprocal interplay between YAP activity in CAFs and tissue stiffness and mechanical stress in the tumor microenvironment are essential for the maintenance of CAF phenotypes and malignancy.^50^

Several studies have shown that nuclear translocation and activation of YAP are associated with Wnt signaling, although the interactions between YAP and Wnt signaling remain complex and intriguing.^33,35,57–59^ Recent studies have also reported that YAP can directly interact with β-catenin. In particular, it was shown that YAP/TAZ is incorporated in the β-catenin destruction complex to dampen the Wnt response.^33^ In our study, we have identified that the loss of YAP in fibroblasts mimics the loss of β-catenin in terms of cell proliferation, migration, and ECM remodeling. Furthermore, we have shown that β-catenin and YAP physically interact in fibroblasts, and YAP nuclear translocation at least partially depends on β-catenin (Fig. 5). The physical interaction between YAP and β-catenin can have interesting consequences with regard to gene transcription. While TCF/LEF1 transcription factors are the canonical nuclear binding partners of β-catenin, and YAP preferentially interacts with TEAD co-factors, YAP/β-catenin binding can coactivate both signaling pathways^60^ and potentially integrate information received from both signaling cascades. There are commonalities in the pathways, i.e., both YAP and β-catenin are regulated by a central cytoplasmic inhibitory complex, and both can be a part of cell adhesion complexes and integrate extracellular signals, making them excellent candidates for cooperation. A recent publication highlighted this cooperation and established an upstream cytoplasmic link between both pathways, WWC family member 3 (WWC3). This dual role of WWC3 has been confirmed in lung cancer and glioma.^61,62^ However, the cooperation of these two crucial pathways in the tumor stroma is still poorly defined. In addition, although YAP’s pro-fibrotic functions have recently been confirmed and linked to interactions with the mesenchymal transcription factor TWIST1,^63^ this and other studies do not address higher complexities of gene expression regulation and cell fate decisions that drive and maintain the fibroblast to CAF transition. For example, the interplay among Hippo, Wnt, and TGF-β signaling, which is the master regulator of the fibrotic process, is rarely explored but is most likely to be crucial for understanding the biology of CAFs.^9^

Understanding the mechanisms of ECM remodeling and its regulation by stromal fibroblasts offers the possibility of developing new therapeutic approaches for melanoma therapy. Collectively, our data suggest that both molecules, β-catenin and YAP, are part of the same signaling axis that controls critical aspects of CAF behavior and that loss of either of them is detrimental to CAF functions. Furthermore, our data demonstrated that melanoma cells and stromal fibroblasts reciprocally interact with each other to regulate ECM remodeling, and this interaction may have a long-lasting effect on the CAF and cancer phenotypes.

## Materials and methods

### Melanoma tissue sections and cells

Human melanoma tissue sections and normal human skin sections were obtained from the Dermatopathology laboratory of the Department of Dermatology at the University of Cincinnati. Mouse *Braf*^*V600E*^*; Pten*^*lox5/lox5*^ melanoma sections were obtained, as previously reported.^14^ Human melanoma cell lines, A375 [*BRAF(V600E)*; *PTEN(mut)*] and SK-MEL-24 [*BRAF(V600E); PTEN(mut)*; *CDKN2A*], were purchased from American Type Culture Collection (ATCC, Manassas, VA) and were maintained in Dulbecco's modified Eagle medium (DMEM) in a humidified incubator at 37 °C with 5% CO_2_. All culture media were supplemented with 10% (v/v) fetal bovine serum (FBS) and 1% (v/v) 10,000 U/ml penicillin and 10,000 U/ml streptomycin (PS) unless otherwise stated. Primary human dermal fibroblasts were isolated from neonatal foreskin in Dr. Kadekaro’s laboratory and were then maintained in DMEM supplemented with 10% (v/v) FBS and 1% (v/v) PS in a humidified incubator at 37 °C with 5% CO_2_. The isolation and maintenance of primary human fibroblasts was approved by the Institutional Review Board and the Institutional Biosafety Office of the University of Cincinnati. All cell culture reagents were purchased from Invitrogen (Carlsbad, CA) unless otherwise stated.

### Generation of inducible β-catenin-deficient human dermal fibroblasts

To ablate β-catenin expression, we transduced primary human dermal fibroblasts with doxycycline-dependent inducible lentiviruses expressing shRNAs that target β-catenin expression (GE Dharmacon, Lafayette, CO). A nontargeting shRNA lentivirus was used to generate control fibroblasts. The inducible lentiviral shRNA vector, which uses the Tet-On inducible system, only allows the expression of either β-catenin-targeting shRNA or a nontargeting shRNA when cells are treated with doxycycline. The expression of GFP is driven by the same tetO promoter so that the transduction and shRNA expression upon doxycycline treatment can be visually tracked by green fluorescence. Briefly, primary human dermal fibroblasts were seeded in 6-well tissue culture plates. When the cells reached 50% confluence, lentiviral particles were added and incubated with the cells for 16 h; then, the medium containing viral particles was replaced with fresh DMEM supplemented with 10% FBS. To select transduced fibroblasts, medium containing 10 μg/ml puromycin was added, and the cells were maintained for three days. Because the inducible lentiviral vector contains a puromycin-resistance gene, transduced cells would survive the selection by puromycin, while fibroblasts without the integration of lentiviral DNA would be killed by puromycin. To evaluate whether puromycin selection was complete, 500 ng/ml doxycycline (Fisher Scientific, Pittsburgh, PA) was added for 48 h to induce the expression of shRNAs and GFP. The percentage of GFP-positive cells was evaluated by flow cytometry as an indication of transduction efficiency and purity of selected fibroblasts. The efficiency of inhibiting β-catenin expression by shRNA was determined by Western blotting and immunostaining as described below.

### YAP knockdown in human stromal fibroblasts

A MISSION® siRNA targeting YAP and a MISSION® siRNA universal negative control were purchased from Sigma-Aldrich (St. Louis, MO). Briefly, human fibroblasts were seeded in a 6-cm culture dish and grown to 60–70% confluency. Next, the culture medium in each well was replaced with 500 µl of DMEM without FBS, and then 25 pmol of siRNAs in 7.5 µl of Lipofectamine® RNAiMAX (Thermo Fisher Scientific, Rochester, NY) was added to the cells, and they were incubated together for six hours at 37 °C. After the incubation, 2 ml of DMEM with 10% FBS was added to each well for incubation overnight. The following day, culture medium with transfection solutions was replaced with regular fibroblast cell culture medium. The cells grew for another 48 h and then were collected for further experimental analysis.

### Labeling human melanoma cells with a red fluorescent protein

To visualize human melanoma cells in 3D spheroids cocultured with GFP-tagged human fibroblasts, we tagged the human melanoma cell line A375 with RFP using a noninducible nontargeting shRNA lentivirus (GE Dharmacon, Lafayette, CO). Lentivirus transduction and selection were carried out as described above. RFP expression and transduction efficiency were confirmed by flow cytometry.

### MTT cell viability assay

In each well of a 24-well plate, 20,000 GFP/Fb or bcat-GFP/Fb were seeded for culture in triplicate after being treated with 500 ng/ml doxycycline for 48 h. At 0, 24, 48, and 72 h, 100 µl of MTT solution (2.5 mg/ml of 3-(4,5-dimethylthiazol-2-yl)-2,5-diphenyltetrazolium bromide in culture medium) was added to each well and incubated for three hours at 37 °C. After incubation, MTT solution and culture medium was carefully removed from each well without disturbing the cells attached at the bottom of the wells. Then, 500 µl of dimethyl sulfoxide (DMSO) was added to each well and incubated at room temperature on a rocking shaker for five minutes. Then, 200 µl of reaction solution from each well was added to a well of the 96-well plate. The absorbance of the solution in each well was read at 594 nm using a microplate reader. Then, 200 µl of DMSO was added to one well of a 96-well plate to be used as a blank. Relative MTT values were calculated using the following formula: relative MTT value = (absorbance of cells − absorbance of DMSO blank)/(absorbance of cells at 0 h − absorbance of DMSO blank at 0 h). A minimum of four wells were prepared for each time point/each cell type. The data are representative of three independent experiments.

### Generation of SK-MEL-24-conditioned culture medium

To generate SK-MEL-24-conditioned medium, SK-MEL-24 melanoma cells were cultured in DMEM with 0.5% FBS for 48 h. After the 48-h culture, the medium was collected for the indicated experiments.

### In vitro scratch assay

GFP/Fb and bcat-GFP/Fb were treated with 500 ng/ml doxycycline for 48 h and subsequently seeded in 24-well tissue culture plates in triplicate at a density of 80,000 cells/well in DMEM supplemented with 10% FBS and 1% PS. Fibroblasts were incubated in a humidified incubator at 37 °C with 5% CO_2_ for one day to allow them to attach and reach 90% confluence. Culture medium was then switched to serum-reduced medium (DMEM supplemented with 0.5% FBS or SK-MEL-24-conditioned DMEM medium supplemented with 0.5% FBS) supplemented with 0.02 mg/ml mitomycin C (Sigma, St. Louis, MO), which inhibited cell proliferation during the procedure. After two days, mitomycin C was removed, and the fibroblast layer was scratched using a p200 pipet tip in the center of each well to form a rectangular cell-free area. Fibroblasts were allowed to migrate for 24–48 h depending on the experimental design. Bright-field images were captured using a Carl Zeiss Axiovert 100 TV inverted microscope at the indicated time points.

### Collagen gel contraction assay

Collagen gel was prepared as described previously.^48^ Briefly, 3 mg/ml of type I collagen (Thermo Fisher Scientific, Rochester, NY), DMEM medium, fetal bovine serum, and 1 N NaOH solution were mixed on ice in a volume ratio of 1:1.65:0.3:0.05 to make a collagen solution with a final concentration of 1 mg/ml. The cells were induced with doxycycline for 48 h. A total of 75,000 cells were resuspended in 1 ml of the 1 mg/ml collagen solution and were then plated in one well of a 24-well tissue culture plate. Collagen gels containing fibroblasts were incubated in a humidified incubator at 37 °C with 5% CO_2_ for 30 min to form solid gels. Subsequently, one ml of fresh DMEM medium that was supplemented with either 0.5% or 10% FBS or SK-MEL-24-conditioned medium was added to each well as indicated. After a 3-day incubation, gels were detached from the edge of each well using an angled needle. Gel contraction was monitored daily for up to three days by taking photographs of each gel using a Nikon digital camera, and the size of gels was measured using ImageJ (NIH, Bethesda, MD).

### Confocal reflection microscopy

GFP/Fb and bcat-GFP/Fb were harvested after induction with doxycycline for 48 h. Three mg/ml type I collagen (Thermo Fisher Scientific, Rochester, NY) was diluted in DMEM (with L-glutamine, w/o HEPES, Thermo Fisher Scientific, MA) and supplemented with 10% FBS to a final collagen gel concentration of one mg/ml, and then the cells were suspended at a final density of 1 × 10^5^ cells/ml. The addition of 1 M NaOH was used to neutralize the gel. Then, the gel was allowed to solidify in a 4-well chamber slide (LAB-TEK, Thermo Fisher Scientific, Rochester, NY) at 37 °C in a humidified incubator with 5% CO_2_ for one hour before one ml of medium supplemented with 10% FBS was added. After a 3-day incubation, collagen fiber distribution was assessed using a Zeiss LSM 710 confocal microscope at ×40 magnification. Images were acquired at a depth of at least 100 μm inside the gel to avoid edge effects. Images of at least four areas of each gel were randomly captured for the 3D reconstruction of the matrix using ImageJ. Briefly, 2D images from each stack were binarized, where the collagen fibers were indicated by black pixels. The binarized images were then used to calculate the fiber thickness, fiber spacing and fiber connectivity using ImageJ with the BoneJ (http://bonej.org/) plugin, and the data were analyzed using GraphPad Prism.

### Atomic force microscopy

Collagen gels were embedded with either GFP/Fb or bcat-GFP/Fb in the wells of a 24-well plate as described above for confocal reflection microscopy. After incubating for three days, the gels were transferred to a petri dish and immersed in DMEM with 10% FBS. The stiffness of the gels was then measured using an atomic force microscope (NanoWizard 4a ver. 6.1.99, JPK Instruments) with a silicon nitride cantilever (CSC37, *k* = 0.3 N/m, MikroMasch), as shown in Fig. 2n. The Young’s modulus was calculated from the force–distance curves using a modified Hertz model of JPK Data Processing (version 6.1.99, JPK Instruments).

### 3D coculture of human melanoma cells and fibroblasts

3D coculture spheroids were formed by mixing 5000 SK-MEL-24 or RFP-tagged A375 melanoma cells with 5000 green fluorescent GFP/Fb or bcat-GFP/Fb in a total volume of 100 μl in 96-well plates with a low cell-adhesion surface. Spheroid formation was initiated by centrifuging the plates at 1000 g for 10 min in DMEM supplemented with 10% FBS, and then incubating the plates at 37 °C in a humidified incubator with 5% CO_2_ for 96 h. Brightfield pictures of spheroids were taken using a Carl Zeiss Axiovert 100 TV inverted microscope with a ×5 objective. Fluorescent images of spheroids were taken using a Zeiss LSM 710 confocal microscope with a ×4 objective. The fluorescence intensities of melanoma cells and fibroblasts were measured and analyzed using ImageJ. To evaluate cell death and apoptosis in spheroids, 50 spheroids from each culture condition were collected and digested using 2 mg/ml collagenase IV (Therma Fisher Scientific, MA) for one hour with stirring to produce a single cell suspension. The cell mixture was stained using an Allophycocyanin (APC) Annexin V Apoptosis Detection Kit (eBioscience, San Diego, CA) and analyzed using a BD FACSCanto flow cytometer (Becton Dickinson, San Jose, CA). SK-MEL-24 melanoma cells were identified as green fluorescence-negative cells since only stromal fibroblasts expressed GFP.

For coculture experiments using RFP-tagged A375, A375 + GFP/Fb spheroids and A375 + bcat-GFP/Fb spheroids were cocultured for 144 h. Pictures were taken using a Zeiss LSM 710 confocal microscope with a ×4 objective; then, the spheroids were collected and processed using 2 mg/ml collagenase (Thermo Fisher Scientific, Rochester, NY, 17018–029) for cell counting based on red and green fluorescence using a Countess II Automated Cell Counter (Thermo Fisher Scientific, Rochester, NY). For fluorescence intensity quantification, fluorescence intensities of 36 A375-GFP/Fb spheroids and 36 A375-bcat-GFP/Fb spheroids were quantified with a Gen5 Microplate Reader and Imager Software using a Cytation 1 imaging multimode reader (Biotek, Winooski, VT).

### Collagen staining

Picrosirius red staining was performed according to the manufacturer’s instructions (American MasterTech, Lodi, CA). Paraffin sections of spheroids were dewaxed, hydrated and stained in picrosirius red dye for one hour. After staining, the slides were washed with 1% acetic acid for one minute, and excessive buffer was removed from the slides by vigorous shaking. Sections were then dehydrated in 100% ethanol, cleared using xylene and mounted with permanent mounting medium (Vector lab, Burlingame, CA).

### Quantification of collagen content

Collagen content was quantified using a Sirius Red/Fast Green Collagen Staining Kit (Chondrex, Redmond, WA) according to the manufacturer's instructions. First, two paraffin sections of spheroids (on one slide) were deparaffinized and transferred to an individual petri dish. A 0.3 ml aliquot of Sirius Red/Fast Green dye solution was added to each petri dish to immerse the sections, and then they were incubated for 30 min at room temperature. After incubation, the dye solution was carefully removed, and the slides were rinsed with tap water until no color was observed in the water. Each slide was subsequently transferred to a new petri dish and covered with 1 ml of dye extraction buffer from the kit. The dye was extracted from the stained samples with gentle shaking and pipetting. Then, 200 µl of eluted dye solution was transferred to a 96-well plate for optical density (OD) reading at 540 nm and 605 nm using a microplate reader. The collagen content in each sample was normalized to the total protein content.

### Immunohistochemistry and immunofluorescence staining

GFP/Fb or bcat-GFP/Fb fibroblasts were first treated with 500 ng/ml doxycycline for 48 h. For fibroblast-only cultures, ten thousand induced cells were counted and seeded in each well of a 4-well Nunc™ Lab-Tek™ II chamber slide (Thermo Fisher Scientific, Rochester, NY). To coculture human melanoma cells and fibroblasts, five thousand induced GFP/Fb or bcat-GFP/Fb were treated with 500 ng/ml doxycycline and were then seeded in one well of a 4-well Nunc™ Lab-Tek™ II chamber slide (Thermo Fisher Scientific, Rochester, NY) with 5000 SK-MEL-24 human melanoma cells. At the end of the coculture, the cells on the slides were fixed in 4% paraformaldehyde for 15 min at room temperature and were then permeabilized using 0.5% Triton-100 for 10 min on ice for immunofluorescence staining. After permeabilization, the cells were washed three times with PBS before blocking with 10% normal goat serum for 1 h at room temperature. Primary antibodies recognizing paxillin (BD Biosciences, 610051, 1:200), fibronectin (Sigma, F3648, 1:200), vimentin (Abcam, ab92547, 1:200), α-SMA (Thermo Fisher, 14–9760–82, 1:200), FSP-1 (Abcam, ab124805, 1:200), YAP (Cell Signaling, 14074, 1:400), and β-catenin (Sigma, MA1–301, 1:1000) were then added to each specific chamber and incubated for two hours at room temperature. An Alexa Fluor 594-conjugated secondary antibody (Invitrogen, Carlsbad, CA) was used to visualize the expression of proteins. Images were taken using a Zeiss LSM 710 confocal microscope, and they were processed using Adobe Photoshop (Adobe, San Jose, CA). F-actin was stained using rhodamine phalloidin (Invitrogen, #R415). Fluorescence intensity per cell was quantified using ZEN 2.3 software. A contour tool in the software was used to circle the area of interest (whole cell or cytoplasm). A minimum of 30 cells per condition were randomly selected and analyzed using GraphPad Prism (GraphPad Software Inc., San Diego, CA).

For cocultured spheroids, collected spheroids were washed once in PBS, fixed overnight in 4% paraformaldehyde, and embedded in agarose gel for the preparation of paraffin blocks. Paraffin sections that were 5-μm-thick were used for immunofluorescence staining. Briefly, paraffin sections were deparaffinized, rehydrated and then unmasked in citrate buffer (pH 6.0) using a microwave heating method. After washing with PBS, sections were blocked in 10% bovine serum albumin (BSA) (Thermo Fisher Scientific, Rochester, NY) in PBS and were subsequently incubated at 4 °C overnight with the respective primary antibody. The following primary antibodies were used: Ki67 (BD Biosciences, 550609, 1:100), fibronectin (Sigma, F3648 1:500), vimentin (Abcam, ab92547, 1:200), α-SMA (Thermo Fisher, 14–9760–82, 1:200) and FSP-1 (Abcam, ab124805, 1:200). The following day, the slides were washed three times using PBS, incubated with the corresponding biotin-conjugated secondary antibodies (Vector Laboratories, Burlingame, CA) at room temperature for one hour, and then incubated with fluorochrome-labeled streptavidin (Vector Laboratories, Burlingame, CA). The slides were mounted with VECTASHIELD Antifade Mounting Medium (Vector Laboratories, Burlingame, CA) and coverslipped. Images were acquired using a Nikon Eclipse 80i fluorescence microscope.

### Coimmunoprecipitation

Coimmunoprecipitation was performed using a Pierce Crosslink Immunoprecipitation Kit according to the manufacturer's instructions (ThermoFisher, Waltham, MA). Briefly, 10 μg of β-catenin antibody (Thermo Fisher, MA1–301, 1 mg/ml) or mouse IgG (ThermoFisher, 31903, 5.6 mg/ml) were added to a spin column containing 20 μl of resin slurry in a 2 ml collection tube, and the mix was incubated at room temperature with gentle rotation for one hour. After incubation, the spin columns were centrifuged, and then the antibody-bound resin was rinsed with ×1 coupling buffer three times to remove unbound antibodies. A 2.5 mM disuccinimidyl suberate (DSS) crosslinker solution was added to crosslink the antibody to the resin. Next, 750 μg of protein extract from either GFP/Fb or bcat-GFP/Fb in a total volume of 500 μl was added to the spin column and rotated end-over-end at 4 °C overnight. After incubation, the column was washed three times with ×1 TBS and one time with ×1 conditioning buffer. Then, 50 μl of elution buffer was used to elute the samples from the column. The eluate was boiled in ×1 sample buffer at 95 °C for 5 min for Western blot analysis.

### Proximity ligation assay

A proximity ligation assay was performed using a Duolink® In Situ Red Starter Kit Mouse/Rabbit per manufacturer's instructions (Sigma-Aldrich, St. Louis, MO). Briefly, fibroblasts were seeded in chamber slides and allowed to grow to 60–70% confluency. The cells were washed twice with PBS and fixed in 3.7% formaldehyde for 10 min at room temperature. Next, the cells were permeabilized using 0.05% Triton X-100 at 4 °C for 15 min, washed, and blocked with Duolink blocking buffer for one hour at 37 °C. The primary antibodies used were mouse anti-β-catenin (ThermoFisher, MA1–301, 1:1000), rabbit anti-YAP (Cell signaling, 14074, 1:200), rabbit anti-K14 (Biolegend, PRB-155P 1:1000), and rabbit anti-N-cadherin (Abcam, ab18230, 1:200), and they each were diluted in Duolink antibody diluent. Diluted primary antibodies were then added to each designated chamber well and incubated overnight with gentle agitation at 4 °C. After incubation, the cells were washed twice at room temperature with wash buffer A, which was supplied in the kit. Two PLA probes (anti-rabbit PLUS and anti-mouse MINUS) were prewarmed at room temperature, mixed with the Duolink antibody diluent and added to the cells for a 1-h incubation at 37 °C. After probe incubation, the cells were washed again with wash buffer A twice for five minutes each. The ligation-ligase solution was then added to each well for an incubation of 30 min at 37 °C so that the two probes could be ligated to form a closed circle template. Next, the cells were washed again with wash buffer A twice for 2 min each. The circle template that formed was amplified by adding polymerase in amplification buffer containing nucleotides and fluorescently labeled oligonucleotides. Distinct fluorescent spots were visualized by fluorescently labeled oligonucleotides in the amplified product. The slides were mounted with Duolink *in situ* mounting medium with DAPI for 30 min, and the images were analyzed using a confocal microscope.

### Western blotting

Western blotting was performed as previously described.^14^ The following antibodies were used: anti-β-catenin (ThermoFisher, MA1–301, 1:2000), anti-F-actin (Abcam, ab205, 1:500), anti-paxillin (BD Biosciences, 610051, 1:2000), anti-vimentin (Abcam, ab92547, 1:1000), anti-fibronectin (Sigma-Aldrich, F3648, 1:2000), anti-YAP (Novus, NB110–58358, 1:200), anti-actin (1:2000, Abcam, ab3280) and anti-GAPDH (Invitrogen, AM4300, 1:1000).

For chemiluminescent Western blotting using X-ray film, the membranes were incubated with either an HRP-conjugated goat anti-mouse IgG (H + L) antibody (Cell Signaling Technology, #7076, 1:2000) or an HRP-conjugated goat anti-rabbit IgG (H + L) antibody (Cell Signaling Technology, #7074, 1:1000) at room temperature for one hour according to the species host of the primary antibody. To ensure equal sample loading, the membranes were incubated with a monoclonal β-actin antibody. X-ray films of the Western blots were scanned, and the density of each band was quantified using ImageJ software. The relative expression level of each detected protein was determined by normalizing the intensity of the target protein band to the intensity of actin bands; the resulting data are presented as the mean ± SEM.

For fluorescent Western blotting using an Odyssey imaging system, membranes were incubated with primary antibodies, washed three times using TBST (Tris-buffered saline, 0.1% Tween 20), and then incubated with IRDye 800CW donkey anti-rabbit IgG secondary antibody (LI-COR, 926–32213, 1:2000) or IRDye 680RD donkey anti-mouse IgG secondary antibody (LI-COR, 926–68072, 1:2000). The membranes were then washed again using TBST and processed for scanning and visualization using an Odyssey CLx imaging system (LI-COR, model #9140). Protein bands were quantified using ImageJ and were analyzed using GraphPad Prism (GraphPad Software Inc., San Diego, CA).

### Wnt ligand and inhibitor assay

In total 10,000 GFP/Fb and bcat-GFP/Fb were seeded in each well of an 8-well chamber slide in DMEM with 0.5% FBS. After 24 h, the cells in dedicated wells were treated with SK-MEL-24 CM, 100 μM ICG-100 (Selleckchem, Houston, TX), 100 μM isoquercitrin (Selleckchem, Houston, TX), and 100 ng/ml WNT3A (R&D, Minneapolis, MN) for 48 h. After 48 h of treatment, the cells in the chamber slide were fixed and immunostained with an anti-YAP antibody (Cell Signaling, 14074, 1:200). For each treatment in the experiments, a minimum of thirty cells from ten random fields was selected for quantifying the fluorescent intensity of nuclear YAP staining using ImageJ. The data are representative of three independent experiments and are presented as the mean ± SEM.

### Quantitative real-Time PCR assay

After 48 h, transfecting with a YAP siRNA or a scramble siRNA, fibroblasts were collected for RNA extraction using a Purelink^TM^ RNA Mini kit (ThermoFisher, Waltham, MA) according to the manufacturer’s protocol. The RNA concentration was determined using a NanoDrop™ spectrophotometer. cDNAs were generated for real-time PCR by reverse transcription using a SuperScript^TM^ IV first-strand synthesis system (ThermoFisher, Waltham, MA). qPCRs were performed using SYPR green master mix power on a StepOnePlus real-time PCR system (Applied Biosystems). qPCR primers for ADAMTS1, COL1A1, COL6A1, TNC, MMP10, MMP13, TIMP2 and TIMP3 were purchased from realtimeprimers.com (Philadelphia, PA). Relative gene expression levels were normalized to GAPDH. The data are representative of three independent experiments.

### Statistical analysis

All data were analyzed using a GraphPad Prism 8 software package (GraphPad Software Inc., San Diego, CA) and are expressed as the mean ± SEM. Differences between means were determined by Student's *t*-tests and were considered statistically significant at *P* < 0.05.

## Supplementary information

Supplementary materials
