# Supplementary material for: The β-catenin/YAP signaling axis is a key regulator of melanoma-associated fibroblasts
Source: Signal Transduct Target Ther. 2019 Dec 24;4:63. doi: 10.1038/s41392-019-0100-7 (PMC6928146; doi:10.1038/s41392-019-0100-7)
Supplement: Supplementary file 1 — Supplementary materials [file 41392_2019_100_MOESM1_ESM.docx]

Supplementary Materials for

**Stromal β-catenin/YAP Axis Is a Key Regulator of Melanoma-Associated Fibroblasts**

Tianyi Liu^1^, Linli Zhou^1^, Kun Yang^1^, Kentaro Iwasawa^4,5^, Ana Luisa Kadekaro^3^, Takanori Takebe^4,5,6,7,8^, Thomas Andl^2^, Yuhang Zhang^1,^*

Correspondence to: yuhang.zhang@uc.edu

**This PDF file includes:**

Figures. S1 to S2


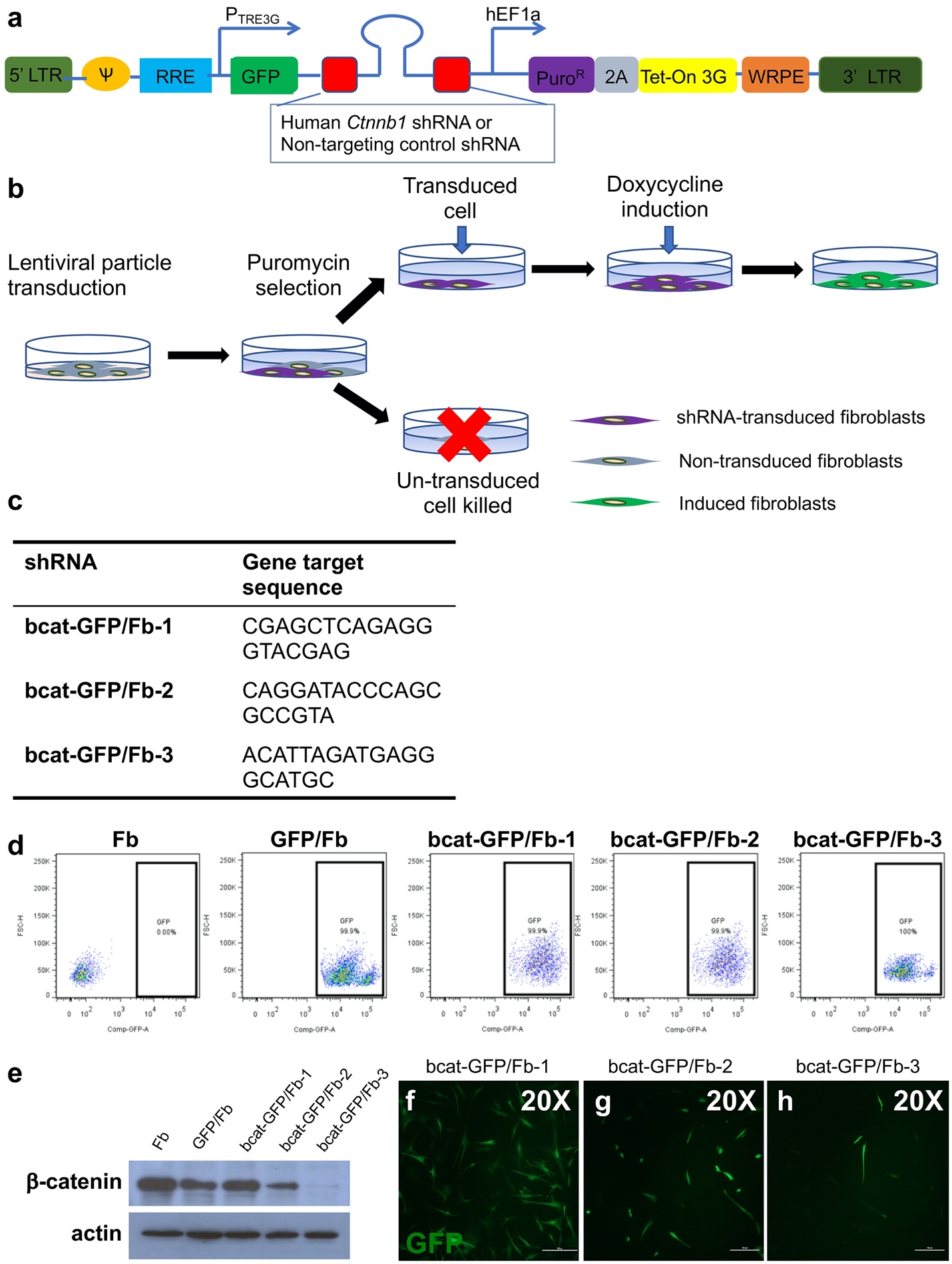


**Figure S1. β-catenin expression is knocked down in primary human dermal fibroblasts using shRNA. a.** Diagram of the lentiviral vector that expresses shRNA to knock down β-catenin expression**.** shRNA targeting β-catenin was inserted into the inducible lentiviral shRNA vector and then packaged into lentiviral particles to infect human dermal fibroblasts. GFP-only fibroblasts (GFP/Fb) were generated using the non-silencing control lentivirus. **b.** Flow chart of lentiviral transduction, puromycin selection, and doxycycline induction to generate desired β-catenin-deficient human adult dermal fibroblasts. **c.** Three different shRNAs were designed to silence β-catenin expression. **d.** Flow cytometry analysis of GFP expression after doxycycline treatment of transduced and puromycin-selected human dermal fibroblasts for 48 hours. Non-transduced human fibroblasts (Fb) were used as a negative control. **e.** β-catenin expression in transduced and selected human fibroblast lines after 48-hour induction by Western blotting. Non-transduced Fb and GFP/Fb were used as positive controls for β-catenin expression. Images shown were representative of at least three independent experiments. **f-h**. Images of GFP expression in bcat-GFP/Fb-1, bcat-GFP/Fb-2 and bcat-GFP/Fb-3. Same number of fibroblasts carrying each shRNA was seeded for comparison. After doxycycline induction for 48 hours, the number of bcat-GFP/Fb-3 was significantly lower than those of bcat-GFP/Fb-1 and bcat-GFP/Fb-2 per field. Images are representative of at least three independent repeats. Scale bar: 100 μm.


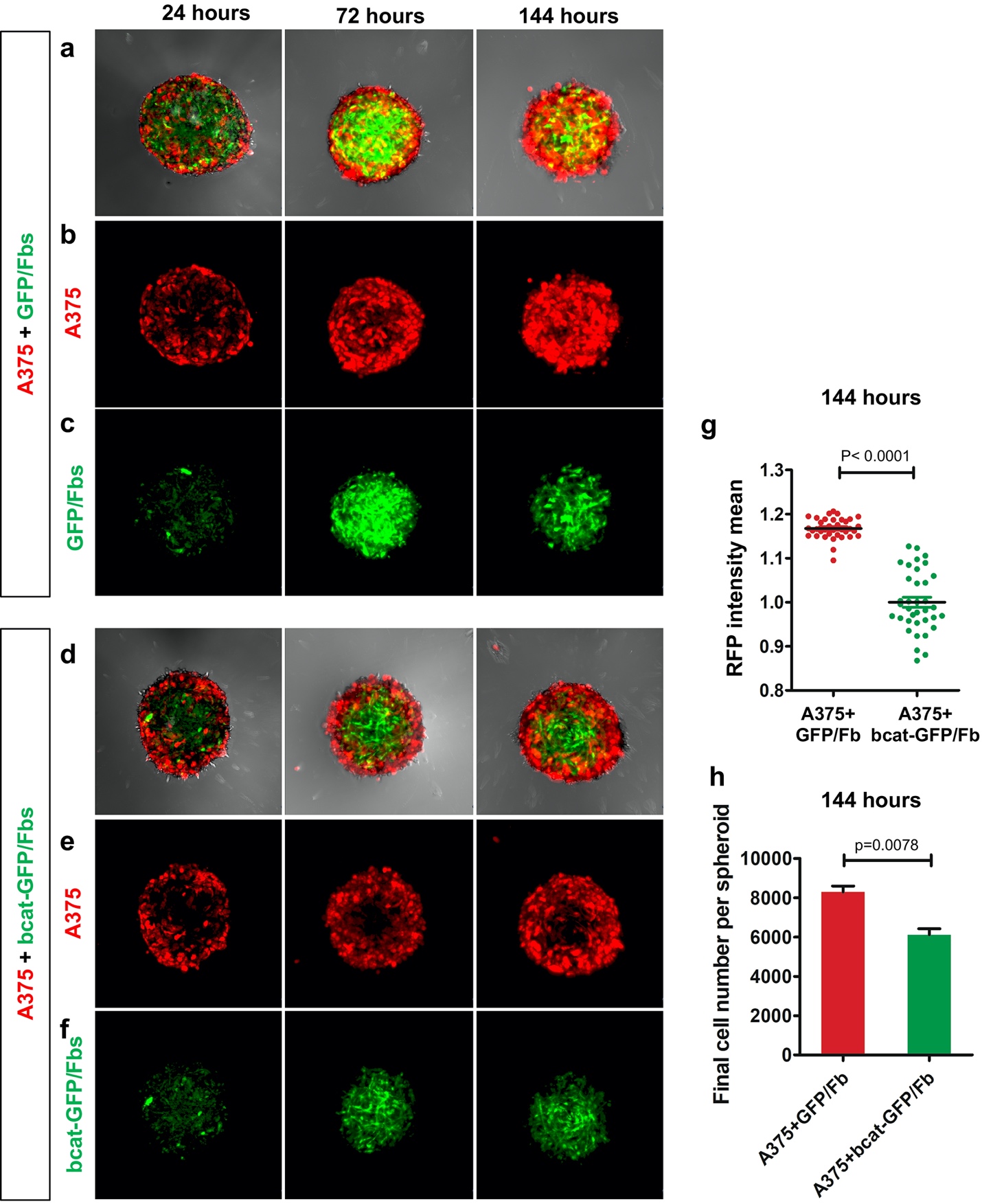


**Figure S2**. **β-catenin is required by stromal fibroblasts to promote A375 melanoma cell growth*.* a**. Representative fluorescent images of A375 + GFP/Fb spheroid at 24, 72 and 144 hours were taken using a multispectral confocal microscope at 4X magnification. **b.** Red fluorescent pictures of A375 + GFP/Fb spheroid at 24, 72 and 144 hours. **c.** Green fluorescent pictures of A375 + GFP/Fb spheroid at 24, 72 and 144 hours. **d**. Representative fluorescent images of A375 + bcat-GFP/Fb spheroid at 24, 72 and 144 hours were taken using a multispectral confocal microscope at 4X magnification. **e.** Red fluorescent pictures of A375 + bcat-GFP/Fb spheroid at 24, 72 and 144 hours. **f.** Green fluorescent pictures of A375 + bcat-GFP/Fb spheroid at 24, 72 and 144 hours. **g**. Quantification of red fluorescence intensity in both groups at 144 hours. **h**. Average numbers of red fluorescent melanoma cells in each A375 + GFP/Fb spheroid and A375 + bcat-GFP/Fb spheroid at 144 hours. A minimum of ten A375 + GFP/Fb spheroids and ten A375 + bcat-GFP/Fb spheroid were counted and analyzed.
